# Elevated levels of soluble RAGE predict impaired alveolar fluid clearance in a translational mouse model of acute respiratory distress syndrome

**DOI:** 10.1186/cc14317

**Published:** 2015-03-16

**Authors:** R Blondonnet, M Jabaudon, G Clairefond, J Audard, D Bouvier, G Marceau, P Blanc, P Dechelotte, V Sapin, JM Constantin

**Affiliations:** 1 CHU Clermont-Ferrand, France; 2 Laroratoire R2D2-EA7281, Clermont-Ferrand, France

## Introduction

Receptor for advanced glycation endproducts (RAGE) is a transmembrane receptor expressed in the lung and primarily located on alveolar type I cells. RAGE is implicated in acute respiratory distress syndrome to alveolar inflammation and, when its soluble form sRAGE is assayed in plasma or pulmonary edema fluid, as a marker of AT I cell injury. Functional activity of AT 1 cells can be assessed by the measurement of the alveolar fluid clearance (AFC) rate [1], but the relationship between sRAGE plasma levels of sRAGE and AFC rates has never been investigated. Our objectives were to report plasma levels of sRAGE in a mouse model of direct acid-induced epithelial injury, and to test their correlation with AFC rates.

## Methods

Forty-one CD-1 mice were divided into two groups: an HCl group underwent orotracheal instillation of hydrochloric acid on day 0, and a group control. Mice were evaluated on days 0, 1, 2 and 4 after a 30-minute period of mechanical ventilation. Blood and lung edema fluid (EF) were sampled. Before initiation of MV, all mice received a tracheal instillation of bovine serum albumin (5%) to detect changes in alveolar protein levels over 30 minutes. Plasma levels of sRAGE and total protein levels were measured. AFC rate values were corrected after measurement of mouse serum albumin in EF.

## Results

**Figure 1:**
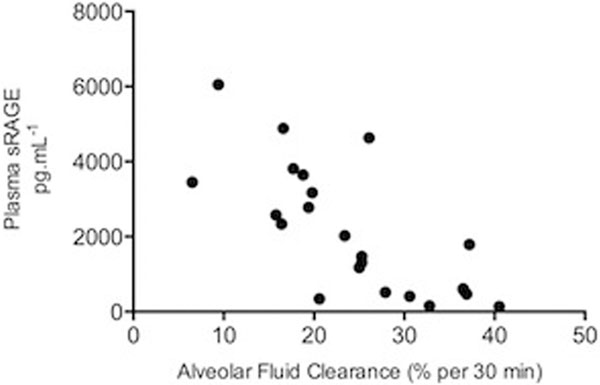
**Relation between AFC rates and sRAGE plasma levels**.

Basal AFC rate was 35% over 30 minutes in HCl-injured mice, but it was significantly depressed on day 1 (16% over 30 minutes; *P *= 0.02). Over time, AFC reached basal levels again. Plasma levels of sRAGE were higher in HCl-treated animals than in control animals on day 1 (*P *= 0.03) and day 2 (*P *= 0.02). The rate of AFC was inversely correlated with sRAGE levels in the plasma (Spearman's ρ = -0.73, *P *< 0.001). See Figure 1.

## Conclusion

The highest impairment in AFC is reported on day 1. sRAGE levels are also higher in injured mice and may be a good surrogate marker of AT I cell injury. This is a newly described relationship between AFC rates and sRAGE plasma level in a mouse model of direct epithelial injury. Our results support further translational investigation on the role of RAGE in alveolar injury and recovery.
